# Obstructive sleep apnea and vitamin D level: Has the dust settled?

**DOI:** 10.1111/crj.13593

**Published:** 2023-02-06

**Authors:** Huai Heng Loh, Norlela Sukor

**Affiliations:** ^1^ Department of Medicine, Faculty of Medicine and Health Sciences Universiti Malaysia Sarawak Kota Samarahan Sarawak Malaysia; ^2^ Department of Medicine, Faculty of Medicine Universiti Kebangsaan Malaysia Medical Center (UKMMC) Kuala Lumpur Malaysia

**Keywords:** apnea hypopnea index, cholecalciferol, continuous positive airway pressure, hypoxia, obesity, sleep disorders

## Abstract

Obstructive sleep apnea and vitamin D deficiency are associated with multiple complications with increased morbidity and mortality. However, the relationship between these two entities remains unclear, with clinical studies demonstrating contradictory results. This narrative review aims to present the current evidence and understanding of this relationship and discuss the possible mechanisms linking these two disease entities. Finally, we summarize and propose areas of opportunity for future research.

## Graphical abstract

The relationship between obstructive sleep apnea and vitamin D deficiency remains unclear. This review presents the current evidence and understanding of this relationship and discuss the possible mechanisms linking these two entities.

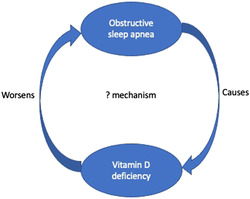

## 1 INTRODUCTION

Obstructive sleep apnea (OSA) is a chronic disease, characterized by recurrent partial or complete upper airway collapse during sleep leading to intermittent hypoxia and sleep disruption.^1^, ^2^ Abrupt oxygen desaturation during sleep leads to brief arousal from sleep in order to terminate the obstruction and restore normal breathing.^3^ This causes substantial sleep fragmentation and impaired sleep quality. The prevalence of OSA has been increasing over the years, especially in developed countries.^4^, ^5^ OSA is commonly found in patients with obesity. This is believed to be due to fat deposits in the upper airway with reduction of muscle activity in that region leading to hypopneic and apneic episodes.^6^ OSA has been demonstrated as an independent risk factor for cardiovascular diseases and is associated with increased cardiovascular morbidity and mortality.^7^, ^8^ This might be contributed by low‐grade inflammation and production of pro‐inflammatory cytokines causing endothelial dysfunction.^9^, ^10^, ^11^ A recent meta‐analysis demonstrated that patients with OSA have higher level of renin‐angiotensin‐aldosterone system hormones, blood pressure, and heart rate compared with those without OSA, which may add on to the increased cardiovascular risks among this cohort.^12^

Although traditionally, vitamin D is thought to play its main role in calcium homeostasis and regulation, there is now increasing evidence that low vitamin D level is associated with a multitude of cardio‐metabolic complications, which has sparked new interests in these extra‐skeletal associations.^13^ Hypovitaminosis D is related to increased risks of cardiovascular diseases, metabolic dysfunctions, worse cardiovascular outcomes, and elevated all‐cause mortality.^14^, ^15^, ^16^, ^17^

Vitamin D levels are associated with respiratory function.^18^ OSA and vitamin D deficiency seem to share common risk factors, such as obesity and increasing age. These two conditions have almost similar pathogenesis, such as involvement of inflammatory reactions and oxidative stress, although the exact mechanism is poorly understood. To date, studies examining the relationship between these two entities have shown contradictory results. The association is likely to be bi‐directional, multi‐factorial, and complex. To better comprehend the relationship between these two entities, this review aims to summarize the current evidence and to present the possible mechanisms and understanding of this association.

## 2 CLINICAL STUDIES OF OSA AND VITAMIN D

### 2.1 Vitamin D level in OSA

Among 139 patients with OSA, vitamin D level was significantly lower compared with 30 non‐apneic cohort (17.78 ± 7.8 vs. 23.9 ± 12.4 ng/mL, *p* = 0.019). However, those with OSA were older and had significantly higher body mass index (BMI), neck, waist, and hip circumferences, which could have contributed to the lower level of vitamin D.^19^ Nevertheless, even among BMI‐matched obese male patients, vitamin D level was still significantly lower among those with OSA compared with those without,^20^, ^21^ suggesting a relationship between OSA and vitamin D deficiency irrespective of weight. In addition, vitamin D level was noted to have inverse correlation with sleep stage transitions, which are indicators of sleep continuity.^19^ Furthermore, the level was demonstrated to be inversely correlated with disease severity even after multi‐variate analysis,^20^, ^22^, ^23^, ^24^, ^25^, ^26^, ^27^ suggesting the role of sleep fragmentation in vitamin D deficiency. This may be the reason why lower level of vitamin D is more pronounced in severe OSA compared with those without OSA.^23^, ^24^

Similarly, the number of patients with vitamin D deficiency was reported to be higher in the OSA group compared with those without OSA.^24^, ^25^ Although few studies found no significant difference in vitamin D level between these two cohorts, these studies still demonstrated that the number of patients with vitamin D deficiency, especially at a level of <10 mcg/dL, was higher in the OSA group, and more pronounced with increasing disease severity, compared with those without OSA.^22^, ^28^, ^29^

### 2.2 Vitamin D level in elderly

The difference in vitamin D level was not apparent among the elderly cohort. Among 72 hospitalized geriatric patients with mild dementia and confirmed OSA, vitamin D level was not significantly different compared with those without OSA (*p* = 0.082).^30^ This could be due to less severe OSA encountered in this study. Nevertheless, the level of vitamin D was demonstrated to reduce with increasing OSA severity (mild OSA 13.5 ng/mL [8.7, 31.2]; moderate OSA 7.9 ng/mL [5.3, 22.6]).

Similarly, in a community‐dwelling elderly cohort with a wide range of BMI, there was no significant difference in the prevalence of vitamin D deficiency. Besides, no association was demonstrated between vitamin D level and apnea hypopnea index (AHI).^31^ Nevertheless, those with lowest quartile of vitamin D concentrations had higher odds of severe sleep apnea. Sensitivity analysis suggested that this association was largely explained by greater BMI and larger neck circumference among men with hypovitaminosis D.^32^

### 2.3 Vitamin D level in metabolic syndrome

Among patients with type 2 diabetes, there was no demonstrated significant difference in vitamin D level between those with OSA and those without (*p* = 0.086).^33^ However, OSA patients with metabolic syndrome had higher prevalence of vitamin D deficiency, which is most pronounced among those with severe metabolic syndrome (metabolic index >3).^34^ They also had significantly lower vitamin D level compared with those without metabolic syndrome (18 ± 8.6 vs. 23.9 ± 14.1 ng/mL, *p* = 0.012).^35^ Low vitamin D level was noted to be associated with increased abdominal obesity, elevated triglyceride level, and reduced HDL‐cholesterol level, as well as diabetes mellitus in this cohort of patients.^36^

### 2.4 Clinical characteristics

OSA patients with vitamin D deficiency were more likely to be females, older, African Americans, with higher BMI, and larger waist circumference compared with those who were vitamin D sufficient.^25^, ^36^ These individuals had concurrent metabolic syndrome and diabetes mellitus. There was no correlation found between vitamin D level and excessive daytime sleepiness.^22^, ^37^ The threshold AHI for vitamin D deficiency was reported to be 19.3 (sensitivity 55.8%, specificity 71.8%),^24^ suggesting that vitamin D deficiency is more pronounced among those with moderate to severe OSA, and the level worsens with OSA severity.

The articles reviewed are summarized in Table 1.

**TABLE 1:** Summary of papers reviewed.

Study	Design	Objectives	Samples, *n*	Methodology	Results	Conclusion
Bozkurt 2012	Cross‐sectional	Vitamin D status of OSA Identify potential links between lower vitamin D levels and abnormal glucose metabolism	143 non‐diabetic OSA versus 47 non‐diabetic non‐OSA Mean age 49.7 (9.8) versus 46.03 (10.8)	Serum 25OHD, HbA1c, insulin levels, and 75‐g OGTT evaluated in all subjects	Serum 25OHD lower among OSA compared with controls; decrement of 25OHD parallel to severity of OSA; severe female OSA lowest serum 25OHD level, male controls highest serum 25OHD level; serum 25OHD of insulin resistant subjects lower than non‐insulin resistant subjects	Vitamin D deficiency may play a role and/or worsen OSA adverse outcomes on glucose metabolism
Mete 2013	Cross‐sectional	Association between serum 25OHD and disease severity in OSA	150 OSA versus 32 non‐OSA Mean age 47.21 (8.7) versus 46.94 (8.1)	Serum 25OHD, PTH, calcium, and phosphorus evaluated in all subjects	No significant difference in serum 25OHD between groups; patients with severe OSA had lower serum 25OHD compared with OSA of other categories and control group; number of patients with vitamin D deficiency higher in OSA group than controls	Vitamin D deficiency more pronounced with increasing OSA severity
Goswami 2016	Cross‐sectional	Determine if lower 25OHD concentration is associated with greater prevalence and increased severity of OSA, independent of established OSA risk factors	2827 community‐dwelling elderly Mean age 76.4 (5.5)	Analysis of serum 25OHD level, demographic and comorbidity data from Outcomes of Sleep Disorders in Older Men study	Subjects with lowest quartile of serum 25OHD had greater odds of severe sleep apnea compared with highest 25OHD quartile but confounded by larger BMI and neck circumference	Association between lower 25OHD and sleep apnea among community‐dwelling elderly largely explained by confounding by larger body mass index and neck circumference
Kerley 2016	Cross‐sectional	25OHD level in OSA and possible relationships to OSA severity, sleepiness, lung function, nocturnal heart rate, and body composition	75 urban Caucasian OSA versus 31 urban Caucasian non‐OSA Median age 54.5	BMI, body composition, neck circumference, sleepiness, lung function, and vitamin D status compared across OSA severity categories and non‐OSA	25OHD inversely correlated with BMI, percent body fat, AHI, and nocturnal heart rate 25OHD independently associated with AHI and nocturnal heart rate 25OHD significantly lower in OSA than non‐OSA	25OHD and OSA are related
Salepci 2017	Cross‐sectional	25OHD level in OSA and identify associated risk factors for vitamin D deficiency	162 OSA versus 19 non‐OSA Mean age 49 (12)	25OHD level evaluated in all subjects	74% of patients had vitamin D deficiency No significant difference in 25OHD level between OSA and non‐OSA and across all OSA severity categories No association between 25OHD level and AHI or BMI	Large proportion of patients referred for OSA evaluation had vitamin D deficiency but did not differ by OSA diagnosis and disease severity
Archontogeorgis 2018	Cross‐sectional	Association between 25OHD with anthropometric and sleep characteristics of OSA and compared with non‐OSA	139 OSA versus 30 non‐OSA Mean age 53.9 (12.8) versus 44.9 (12.8)		Serum 25OHD level lower in OSA than non‐OSA In OSA, 25OHD level negatively correlated with sleep stage transitions, AHI, oxygen desaturation index, percentage of time with oxyhemoglobin saturation <90%, positively correlated with average oxyhemoglobin saturation during sleep, forced expiratory volume in 1 s, and oxygen partial pressure	25OHD level lower in OSA and correlated with indices of OSA severity
Archontogeorgis 2018	Cross‐sectional	25OHD level according to presence of MetS and its components in OSA	55 OSA with MetS versus 52 OSA without MetS Mean age 54.8 (12) versus 51.8 (13.5)	25OHD level evaluated in all subjects	25OHD level lower among OSA with MetS compared with OSA without MetS 25OHD level lower in OSA with higher metabolic score	OSA with MetS has lower 25OHD level compared with OSA without MetS
Pazarli 2018	Cross‐sectional	Association between bone mineral density and 25OHD level in OSA	75 OSA versus 21 non‐OSA Mean age 48.55 (11.8)	BMD and 25OHD level evaluated in all subjects	No significant difference in BMD and 25OHD level in OSA severity categories No correlation between sleep indices and BMD parameters	No relationship between OSA and BMD values
Qiao 2018	Cross‐sectional	Relationship between OSA severity and bone metabolic markers	87 OSA (32 mild‐to‐moderate, 55 severe) versus 32 obese non‐OSA Mean age 51.8 (8.1) versus 48.2 (9.9) versus 50.1 (7.3)	BMD, t‐P1NP, N‐MID, beta‐CTX, 25OHD, and PTH evaluated in all subjects	No significant differences in BMD Bone markers higher in severe OSA than control 25OHD lower in OSA than control; level decreased as OSA severity increased Serum PTH higher in severe OSA then mild‐to‐moderate OSA and control AHI correlated with t‐P1NP and PTH Minimum oxygen saturation level correlated with 25OHD and PTH	Bone markers higher in severe OSA, severity of OSA correlated with bone metabolic markers
Ragia 2018	Cross‐sectional	Impact of VDR gene polymorphic variation on 25OHD concentration and susceptibility to OSA	144 OSA versus 32 non‐OSA Mean age 53.2 (12.4) versus 47.6 (14.3)	Human genetic variation in VDR characterized in all subjects	FokI CC genotype frequency higher in OSA than controls VDR FokI polymorphism explained 14.5% of 25OHD concentration variability and associated with excessive daytime sleepiness	VDR FokI polymorphism associated with vitamin D concentration in OSA Interaction of vitamin D concentration with VDR FokI polymorphism associated with OSA adjusted for other risk factors
Gronewold 2019	Cross‐sectional	Prevalence and severity of sleep disordered breathing in mild dementia and associations with severity of impairment in cognition, emotional function, and mobility	101 elderly with dementia Mean age 84.1 (6.5)	Daytime sleepiness, medical characteristics, cognition, emotional function, and mobility assessed in all subjects	Patients with AHI ≥ 15/h often presented with heart failure and vitamin D deficiency	
Kirac 2019	Cross‐sectional	Association between VDR, VDBP mutations, vitamin D level, and risk factors with OSA	50 OSA versus 50 non‐OSA Mean age 48.82 (11.03) versus 45.86 (8.51)	VDR and VDBP mutations investigated with qPCR	CA genotype in VDBP, CC and AA genotypes in VDR were significant in OSA	VDR and VDBP mutations highly related with OSA
Bouloukaki 2020	Cross‐sectional	25OHD levels in OSA and possible correlations with clinical and PSG parameters	617 OSA versus 68 non‐OSA Mean age 54 (15)	25OHD level determined in all subjects	OSA patients lower vitamin D levels than controls Lowest levels of vitamin D and higher prevalence for vitamin D deficiency in severe OSA Severe OSA independent association with risk of vitamin D deficiency	Large proportion of patients referred for OSA evaluation had vitamin D deficiency and independently associated with severe OSA
Ma 2020	Cross‐sectional	Association of 25OHD level with severity of OSA in T2D	106 OSA and T2D versus 30 T2D non‐OSA Mean age 49.0 (14.0)	25OHD level determined in all subjects	No significant differences in 25OHD in all OSA severity categories 25OHD level not correlated with AHI or risk of OSA	25OHD level not associated with AHI or risk of OSA in T2D
Siachpazidou 2020	Prospective	25OHD level in OSA and changes after 3 and 12 months of CPAP	30 OSA versus 30 non‐OSA Mean age 50.3 (13.8) versus 56.1 (8.1)	25OHD level at baseline, 3 and 12 months of CPAP in OSA	No significant difference in 25OHD between OSA and control No change in 25OHD after 3 and 12 months of CPAP CPAP‐adherence patients less reduction in 25OHD compared with non‐adherent patients after 1 year 25OHD level correlated with higher daily CPAP usage at 3 and 12 months	Good CPAP adherence and high daily CPAP usage positively affected 25OHD level in OSA
Bhatt 2021	Cross‐sectional	Significance of vitamin D, PTH, VDR, PTH gene polymorphisms with body composition, and biochemical investigations in Asian Indian OSA	120 OSA and obese versus 110 obese non‐OSA versus 70 non‐obese non‐OSA Mean age 43.5 (10.6) versus 42.6 (9.6) versus 42.6 (8.6)	VDR and PTH genotyping with qPCR Clinical, body composition, anthropometry, and biochemical investigations	OSA and obese lower 25OHD level, higher PTH level Indirect correlation between 25OHD level and OSA severity VDR and PTH genes significantly associated with OSA VDR haplotype combination variants more frequent in OSA and obesity	Lower 25OHD level in OSA and correlate with disease severity VDR and PTH mutations highly related with OSA in Asian Indians
Sadaf 2021	Prospective	Effect of OSA on BMD and serum 25OHD level	59 OSA versus 34 non‐OSA Mean age 48.02 (4.35) versus 46.35 (7.29)	BMD and 25OHD level assessed in all subjects	Lower BMD and 25OHD level in OSA than control Negative correlation between AHI and BMD, AHI and 25OHD level	OSA affects BMD

Abbreviations: 25OHD, 25‐hydroxyvitamin D; AHI, apnea hypopnea index; beta‐CTX, beta‐C‐terminal telopeptide of type 1 collagen; BMD, bone mineral density; BMI, body mass index; CPAP, continuous positive airway pressure; MetS, metabolic syndrome; Mod, moderate; N‐MID, N‐terminal midfragment of osteocalcin; OGTT, oral glucose tolerance test; OSA, obstructive sleep apnea; PSG, polysomnography; PTH, parathyroid hormone; T2DM, type 2 diabetes mellitus; t‐P1NP, total procollagen type 1 N‐terminal propeptide; VDD, vitamin D deficiency; VDR, vitamin D receptor; VDBP, vitamin D binding protein.

### 2.5 Outcome of continuous positive airway pressure (CPAP) treatment on vitamin D level

Although treatment with CPAP among patients with moderate to severe OSA for 12 weeks did not significantly alter vitamin D level, some changes were seen at 24 weeks among those with severe OSA and with excessive sleepiness.^38^ This suggests that CPAP may have late beneficial effect and exerts more benefit on vitamin D level particularly among those with severe OSA. Nevertheless, the median vitamin D level of this cohort falls within the sufficient range of 50.9 ng/mL, which may explain the non‐significant difference seen with CPAP treatment.

Further improvement of vitamin D level was demonstrated among male OSA patients who initially responded well to a year of adequate CPAP therapy usage.^39^ These findings were consistent with another study that demonstrated that vitamin D levels were positively correlated with higher daily CPAP usage, especially among those who were adherent to CPAP therapy.^40^, ^41^ This suggests that improving hypoxia by normalizing nocturnal oxygen saturation may positively affect vitamin D level.

The studies examining effect of CPAP treatment on vitamin D level among patients with OSA are summarized in Table 2.

**TABLE 2:** Effect of CPAP on 25OHD level among OSA.

Study	Samples, *n*	CPAP duration	Findings
Haglow 2018	34 Mean AHI 39.9	24 weeks	Significant improvement of 25OHD
Liguori 2015	90 Mean AHI 49.7	Seven nights	Significant improvement of vitamin D level in male OSA responders (residual AHI < 5 and CPAP usage >4 h per night)
Liguori 2017	39 Mean AHI 47.8	1 year (extension study from Liguori 2015)	Significant improvement of 25OHD Obese OSA more frequently shifted from vitamin D level <20 to >20 ng/mL compared with non‐obese OSA Positive association between change in vitamin D level and BMI
Siachpazidou 2020	30 Mean AHI 40.4	1 year	No significant improvement of 25OHD Patients with good CPAP adherence had higher 25OHD after 1 year compared with non‐adherent group Vitamin D level correlated with higher daily CPAP usage at 3 and 12 months

Abbreviations: 25OHD, 25‐hydroxyvitamin D; AHI, apnea hypopnea index; BMI, body mass index; CPAP, continuous positive airway pressure; Mod, moderate; OSA, obstructive sleep apnea.

### 2.6 Outcome of vitamin D supplementation on OSA severity

The data of vitamin D supplementation use in patients with OSA are limited. There are only two studies identified, and both were limited by small sample sizes. Among 19 male patients with mild OSA who were not on CPAP, the use of vitamin D3 at 50 000 IU a week for total of 8 weeks significantly reduced OSA severity in terms of oxygen desaturation index, AHI, hypopnea index, and the number of OSA patients.^42^ The second study evaluated the use of vitamin D3 at 4000 IU per day (*n* = 10) versus placebo (*n* = 9) for 15 weeks among patients with OSA of different severity categories and heterogenous CPAP usage. Significant improvement in LDL and lipoprotein‐associated phospholipase A2 was observed; however, post‐intervention OSA severity was not assessed.^43^ In this respect, more robust studies are needed to study the effect of adequate vitamin D supplementation in patients with OSA.

## 3 POSSIBLE MECHANISMS

### 3.1 OSA causing vitamin D deficiency

#### 3.1.1 Sleep duration and sun exposure

Sun exposure is essential in initiation of cutaneous vitamin D synthesis. However, patients with OSA experience nocturnal hypoxia and sleep fragmentation, which may lead to daytime drowsiness and fatigue. This may cause reduction in outdoor activities, leading to lack of sun exposure with subsequent reduction in vitamin D synthesis.^44^ Indoor workers are consistently reported to experience vitamin D insufficiency or deficiency, suggesting the lack of exposure to ultraviolet light leading to reduction in vitamin D level.^45^ In addition, short sleep duration of less than 6 h a night secondary to sleep fragmentation was reported to be associated with lower level of vitamin D. These individuals have twice increased odds of having vitamin D deficiency, independent of age, gender, seasonality, BMI, and ethnicity.^46^, ^47^

On the other hand, vitamin D deficiency, especially at a level of <20 ng/mL (<50 nmol/L), was shown to be associated with increased risk of sleep disorders, including poor sleep quality, shorter sleep duration, and sleepiness,^48^ which could contribute to worsening of OSA. The use of vitamin D supplementation among patients with sleep disorders led to significant improvement of sleep quality, reduction of sleep latency, and increment of sleep duration.^49^, ^50^ This further strengthens the relationship between vitamin D deficiency and sleep disorders.

Older adults generally have lower vitamin D levels as old age is an independent risk factor for vitamin D deficiency.^51^, ^52^, ^53^, ^54^ The decline of vitamin D level in the aging process is associated with reduction in skin production of vitamin D, decreased vitamin D receptor (VDR), and reduced ability of renal production of active vitamin D.^55^ This may explain the non‐significant difference seen between those with OSA and those without among the elderly.

#### 3.1.2 Hypoxia

The association between vitamin D deficiency and OSA is also believed to be related to hypoxia involving hypoxia‐inducible factor 1‐α (HIF1‐α).^40^ HIF1‐α is the main factor for oxygen metabolism homeostasis, and its expression is shown to increase in OSA.^56^ The use of vitamin D3 reduced protein expression, transcriptional activity, and target genes of HIF1‐α in various human cancer cells,^57^ substantiating the relationship between vitamin D deficiency and hypoxia. This is further proven with clinical studies, which demonstrated improvement of vitamin D level with attenuation of hypoxia in patients with OSA treated adequately with CPAP therapy.^38^, ^39^, ^40^, ^41^

#### 3.1.3 Obesity

A high proportion of patients with OSA are overweight or obese. Observational studies have demonstrated the relationship between low vitamin D level and obesity to be bi‐directional.^58^ Vitamin D deficiency increases risk of obesity, whereas obesity lowers vitamin D level. Being fat‐soluble, vitamin D is predominantly stored in adipose tissue. Moreover, VDR and the enzymes involved in producing the active form of vitamin D are also expressed in these adipose tissues.^40^ Vitamin D is thus believed to be trapped in the adipose tissue, leading to reduced bioavailability and hence low level of vitamin D in the blood.^59^ This is also contributed by an increased catabolism of vitamin D by local action of 24‐hydroxylase enzyme found in human adipose tissue.^27^ In contrast, some believe that volumetric dilution of vitamin D, instead of sequestration, in the large adipose stores was the reason leading to low serum vitamin D level.^60^ Nevertheless, both theories suggest that obesity plays an important role in vitamin D deficiency in patients with OSA. This is worsened by chronically raised abdominal pressure seen in those who are obese, which may also lead to gastro‐esophageal reflux and gastric ischemia, thereby affecting vitamin D absorption causing vitamin D deficiency.^27^, ^61^

Leptin is a type of adipokine that is a pro‐inflammatory factor predominantly formed by adipose cells. Increment of adipose tissue volume seen in obesity leads to adipocyte hypertrophy, which subsequently causes increment of leptin production.^62^ High levels of leptin were shown to impair vitamin D metabolism by attenuating gene expression responsible for the activation of vitamin D.^63^

On the other hand, the active form of vitamin D modulates adipogenesis and regulates adipocyte differentiation by binding to the nuclear VDR with high affinity.^64^ Hence, a low vitamin D level causes adipose tissue dysregulation leading to obesity and thus increases the risk of OSA.^65^ The use of active vitamin D in both animal and human tissues inhibited adipocyte differentiation,^64^ depicting the role for low vitamin D status in development of obesity.

#### 3.1.4 Metabolic syndrome

Vitamin D level is inversely associated with the presence of metabolic syndrome.^66^, ^67^ Low vitamin D status could magnify the adverse effects of obesity on the metabolic variables, including insulin resistance and hypertension.^36^, ^68^ Vitamin D‐deficient rats exhibited close to 50% reduction in insulin secretion compared with those which were replenished with activated vitamin D.^69^ Pancreatic β cells express VDR, and the activated form of vitamin D is shown to stimulate insulin secretion.^70^ Some longitudinal and observational studies have also demonstrated that low levels of vitamin D predict the risk of type 2 diabetes in Europeans, African Americans, South Asians, and native American children, suggesting that the evolution of diabetes may be influenced by low vitamin D level.^71^, ^72^, ^73^ This may explain why there is no difference seen in vitamin D level between diabetic patients with and without OSA, as vitamin D deficiency is commonly seen in patients with type 2 diabetes regardless of age, gender, and insulin treatment.^33^

Besides, vitamin D and VDR are demonstrated to be directly involved in the modulation and inflammatory pathways leading to the development of metabolic‐associated fatty liver disease (MAFLD), especially among the overweight and obese cohort.^74^ This occurs via liver homeostasis, intra‐hepatic regulation of insulin sensitivity, fat accumulation, and gut homeostasis.^74^ On the other hand, liver disease also impairs protein synthesis and reduces production of vitamin D binding protein (VDBP), leading to a decreased total vitamin D level.^75^ As patients with OSA and metabolic syndrome are at higher risk of MAFLD, the reduction of vitamin D synthesis and VDBP could contribute to development of vitamin D deficiency.^76^, ^77^

The development of chronic kidney disease from the presence of concurrent diabetes and hypertension may also influence vitamin D status and function.^78^ With deteriorating kidney function, there is a slow progressive decline in active vitamin D level due to reduction in renal mass, decreased glomerular filtration rate, and effect of fibroblast growth factor‐23 on the synthesis of active vitamin D.^79^ The transport capacity for VDBP from the glomerular filtrate into the renal tubules is similarly reduced in chronic kidney disease.^75^

#### 3.1.5 Autonomic dysfunction

In healthy individuals, sympathetic neural activity decreases with a concurrent rise in parasympathetic activity during sleep.^80^ However, in patients with OSA, upper airway obstruction and hypopnea are postulated to cause autonomic dysfunction,^81^ with abnormal parasympathetic activity persisting beyond sleep.^82^ As vagal nervous system plays a major role in gastrointestinal motility, abnormal parasympathetic activity in patients with OSA is believed to cause reduction in gastrointestinal motility and gastrointestinal hormone secretion leading to reduced vitamin D absorption.^44^, ^83^

### 3.2 Vitamin D deficiency worsening OSA

#### 3.2.1 VDR gene polymorphism

VDRs are widely distributed throughout many tissues, including the brain regions, which are involved in sleep regulation.^84^, ^85^ Patients with OSA were found to have higher frequency of VDR *FokI* CC genotype, which was associated with lower vitamin D level, compared with non‐OSA controls.^86^ In logistic regression analysis, the interaction of vitamin D with VDR *FokI* polymorphism was associated with higher risk of OSA occurrence after adjustment for various risk factors.^86^ Furthermore, VDR *FokI* polymorphism could affect severity of OSA symptoms. A higher frequency of VDR *FokI* CC genotype was found in OSA patients with excessive daytime sleepiness.^86^ Nevertheless, VDR activity may be confounded by ethnic variation.^86^, ^87^ In Asian and African populations, those with TT genotype were the ones associated with lower vitamin D level.^88^, ^89^, ^90^, ^91^ The underlying mechanism of this difference remains unclear.

#### 3.2.2 Vitamin D and skeletal muscle

Vitamin D plays a role in active calcium transportation into muscle via Ca‐ATPase, as well as regulating muscular contractions.^92^ Muscle weakness is one of the prominent features of vitamin D insufficiency. Chronically low vitamin D level may cause non‐inflammatory myopathy of upper airway muscle due to impaired cellular calcium transportation into the sarcoplasmic reticulum and mitochondria.^84^ This leads to reduced pharyngeal patency and predisposes patients to apneic events during sleep.^93^ Besides, vitamin D deficiency is reported to increase the risk of nasal airflow restriction,^84^ hence further worsening sleep apnea.

#### 3.2.3 Inflammatory cytokines

Chronic variations in vitamin D levels also affect humoral mechanisms as vitamin D harbors immuno‐modulatory properties.^94^ A deficient in this vitamin causes immune dysregulation leading to a rise in inflammatory cytokines, including TNF‐α, which is shown to affect sleep architecture by enhancing slow‐wave sleep.^84^, ^95^ In healthy women, this cytokine was demonstrated to be inversely related to serum vitamin D level.^96^ Interleukin‐17 (IL‐17), a pro‐inflammatory cytokine, was also found to be significantly elevated in patients with severe OSA compared with non‐OSA controls.^97^ A negative correlation was demonstrated between IL‐17 and vitamin D level among those with severe OSA.^97^ Additionally, people who have inadequate vitamin D are found to have increased risk of infection and inflammation of upper and lower airway.^84^ This can cause adeno‐tonsillar hypertrophy, which can worsen airway obstruction in patients with OSA.

**FIGURE 1:**
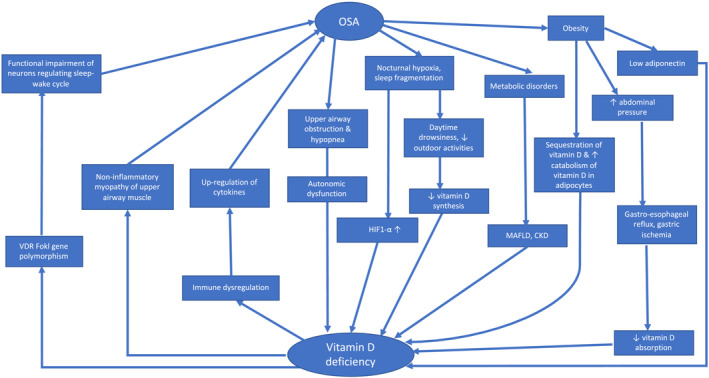
Relationship between obstructive sleep apnea and vitamin D deficiency. CKD, chronic k; HIF1‐α, hypoxia‐inducible factor 1‐alpha; MAFLD, metabolic‐associated fatty liver disease; OSA, obstructive sleep apnea; VDR, vitamin D receptor.

The proposed mechanisms of the bidirectional relationship are summarized in Figure 1.

## 4 RESEARCH GAPS AND FUTURE DIRECTION

Despite the comprehensive summary of the clinical studies and possible mechanisms linking OSA and vitamin D deficiency, there remains gaps in the current literature in this area. Both of these increasingly common diseases are closely related to increased cardio‐metabolic risks with increased morbidity and mortality.^98^, ^99^ It remains unclear whether vitamin D deficiency is a risk factor for OSA or whether OSA is a risk factor for vitamin D deficiency, and the role of other confounding factors in these two disease entities. As clear relationship between OSA and vitamin D deficiency is yet to be established, larger prospective studies to examine the link between the two are needed. This is necessary to better understand the relationship, mechanism of vitamin D deficiency in patients with OSA, and the correlation between vitamin D levels and severity of OSA.

The effect of VDR and VDBP gene polymorphism in OSA remains unclear. Hence, research is needed to examine the role of VDR and VDBP genetic variants and possibly their mutations in OSA severity and OSA‐related metabolic disorders. Inter‐ethnic variations in this genetic polymorphism and mutations are also needed to understand the difference observed in the *FokI* genotype in different ethnic groups.

Other plausible mechanisms that warrant more robust future research include inflammatory pathways involved, associations with adipokines, and the role of autonomic dysfunction linking OSA and vitamin D deficiency.

Furthermore, the use of vitamin D supplement in the OSA cohort is very limited. Hence, prospective studies with bigger sample sizes are essential to determine the effect of vitamin D, as well as the dose and duration needed to bring benefits to patients with OSA. There is a lack of evidence in the role of vitamin D in improving OSA severity; whether vitamin D supplementation would alter the course of OSA, as well as its related metabolic disturbances, such as diabetes mellitus, hypertension, and dyslipidemia remains unanswered. Although current evidence suggests beneficial effects of CPAP therapy on vitamin D levels in patients with OSA, long‐term studies are lacking to better understand the benefits of CPAP on cardiovascular morbidity and mortality outcomes beyond improvement of vitamin D levels in these patients.

## 5 CONCLUSION

The present review aimed to collect and put into perspective current available literature regarding the association between OSA and vitamin D level. It focused on the potential relationship between these two entities and presented evidence for potential causal links and underlying mechanisms. Current evidence suggests a relationship between OSA and low levels of vitamin D via inflammatory and non‐inflammatory pathways, genetic polymorphisms of VDR and VDBP, and autonomic nervous system. The coexistence of obesity and hence increased adipose tissue contributes to the sequestration and catabolism of vitamin D and impaired adipokine function. Hypoxia secondary to sleep fragmentation leading to reduced outdoor activities and sun exposure may play a role linking these two disease entities as well. The presence of liver and kidney disease associated with metabolic disorders seen in these patients can lead to lower vitamin D level. Nevertheless, further robust prospective studies with larger sample sizes are needed to examine this link and the long‐term beneficial effect of OSA‐directed therapy in increasing vitamin D level. It is essential to determine the role of vitamin D supplementation in improving OSA severity and altering the course of OSA. Because untreated OSA and vitamin D deficiency independently lead to increased cardiovascular morbidity and mortality, early recognition through effective screening and diagnosis and a timely targeted treatment are necessary to reduce the risk of adverse sequelae related to OSA and vitamin D deficiency.

## AUTHOR CONTRIBUTIONS

Huai Heng Loh conceived and designed the work; Norlela Sukor revised it critically for important intellectual content. Both authors read and approved the final version of the manuscript and agree to be accountable for all aspects of the work.

## CONFLICT OF INTEREST

All authors certify that they have no affiliations with or involvement in any organization or entity with any financial interest in the subject matter or materials described in this manuscript.

## ETHICS APPROVAL/PATIENT CONSENT

This article does not contain any studies with human participants performed by any of the authors.

## Data Availability

Data sharing is not applicable to this article as no datasets were generated or analyzed during the current study.
